# Expansion and Divergence of Argonaute Genes in the Oomycete Genus *Phytophthora*

**DOI:** 10.3389/fmicb.2018.02841

**Published:** 2018-11-30

**Authors:** Stephanie R. Bollmann, Caroline M. Press, Brett M. Tyler, Niklaus J. Grünwald

**Affiliations:** ^1^Horticultural Crop Research Unit, Agricultural Research Service, United States Department of Agriculture, Corvallis, OR, United States; ^2^Department of Botany and Plant Pathology, Center for Genome Research and Biocomputing, Oregon State University, Corvallis, OR, United States

**Keywords:** *Phytophthora*, argonaute, Chromalveolate, evolution, small RNA, stramenopiles

## Abstract

Modulation of gene expression through RNA interference is well conserved in eukaryotes and is involved in many cellular processes. In the oomycete *Phytophthora*, research on the small RNA machinery and function has started to reveal potential roles in the pathogen, but much is still unknown. We examined Argonaute (AGO) homologs within oomycete genome sequences, especially among *Phytophthora* species, to gain a clearer understanding of the evolution of this well-conserved protein family. We identified AGO homologs across many representative oomycete and stramenopile species, and annotated representative homologs in *P. sojae*. Furthermore, we demonstrate variable transcript levels of all identified *AGO* homologs in comparison to previously identified Dicer-like (DCL) and RNA-dependent RNA polymerase (RDR) homologs. Our phylogenetic analysis further refines the relationship of the AGO homologs in oomycetes and identifies a conserved tandem duplication of *AGO* homologs in a subset of *Phytophthora* species.

## Introduction

The RNA interference (RNAi) machinery, including Argonaute (AGO) proteins, is well conserved among most eukaryotic supergroups (Cerutti and Casas-Mollano, 2006). After small RNA duplex formation by Dicer/Dicer-like (DCR/DCL) enzymes with or without the function of RNA-dependent RNA polymerases (RDR), the duplex is loaded onto an AGO, unwound, and the guide strand is retained to complete the mature RNA-induced silencing complex (RISC) (Schwarz et al., 2003). The AGO effector present in the RISC complex determines whether the target mRNA will be down-regulated through cleavage or translational repression. AGO homologs share a common set of domains, including the N-terminal, Linker 1, PAZ (Piwi Argonaute and Zwille), Linker 2, Mid, and PIWI (originally P-element Induced WImpy testis in *Drosophila*) domains. The N-domain has been shown to be required for unwinding of the small RNA duplex in the process of forming the mature RISC complex (Kwak and Tomari, 2012) and in some AGOs it is required for cleavage of the target mRNA (Hauptmann et al., 2013). The PAZ domain binds and anchors the 3’ end of the small RNA guide (Lingel et al., 2003, 2004; Song et al., 2003; Yan et al., 2003; Ma et al., 2004) and aids in unwinding the small RNA duplex (Gu et al., 2012). The MID domain binds the 5′ nucleotide of the guide sRNA (Frank et al., 2010, 2012), which, in addition to the structure of the small RNA duplex, is important for the binding specificity seen in some AGOs (Tomari et al., 2007; Jannot et al., 2008; Czech et al., 2009; Okamura et al., 2009; Ghildiyal et al., 2010). The MID-PIWI interface binds and anchors the 5′ phosphate of the guide sRNA (Parker et al., 2005). The PIWI domain is the catalytic domain that allows some AGOs to cleave the target mRNA (slicer activity) which is complementary to the bound guide sRNA (Song et al., 2004; Rivas et al., 2005). The catalytic tetrad (DEDD/H) is required, but not sufficient for slicer activity (Liu et al., 2004; Song et al., 2004; Rivas et al., 2005; Nakanishi et al., 2012; Faehnle et al., 2013; Hauptmann et al., 2013; Sheng et al., 2014).

The number and type of AGO homologs varies from organism to organism (Tolia and Joshua-Tor, 2007). There are four major families of AGO homologs: AGO-like, PIWI, WAGO, and *Trypanosoma* AGO families (Yigit et al., 2006; Garcia Silva et al., 2010). The AGO-like family is widely conserved among eukaryotes, is predominantly responsible for interaction with micro (miRNAs) and short interfering RNAs (siRNAs), and is involved in regulation of transcription and translation, maintenance of germ cells, alternative splicing, and heterochromatin formation (Azzam et al., 2012; Michalik et al., 2012; Wei et al., 2012; Gagnon et al., 2014; Huang and Li, 2014). The PIWI family is also well conserved, interacts with PIWI-interacting RNAs (piRNAs), and is involved with germline stem cell maintenance and transposon silencing in stem cells (Lin and Spradling, 1997; Brennecke et al., 2007). Worm-specific AGO (WAGO) proteins are limited to nematodes, and act as secondary Argonautes in response to an initial AGO targeted cleavage of an mRNA, resulting in a more specific silencing response (Yigit et al., 2006). The AGO family in *Trypanosoma*, typified by *T. brucei*, inhibits transposon activity (Djikeng et al., 2001; Shi et al., 2009).

Research on the small RNA biology in the Stramenopile kingdom, including brown algae, diatoms, and oomycetes, is currently limited. The genus *Phytophthora* contains prominent oomycetes such as the Irish famine pathogen *P. infestans* or the sudden oak death pathogen *P. ramorum*, affecting many hosts and leading to multibillion dollar crop damage and devastation of natural environments (Tyler, 2007; Fry, 2008; Grünwald et al., 2008). With the advancement of genome sequencing technologies, several oomycete genomes have become available for comparative genomic analyses (Tyler et al., 2006; Haas et al., 2009; Baxter et al., 2010; Lévesque et al., 2010; Links et al., 2011). Within the Chromalveolate supergroup, putative miRNAs have been described in the apicomplexan *Toxoplasma gondii* (Braun et al., 2010) and a miRNA family was found to be conserved across three diverse *Phytophthora* genomes, with evidence for target cleavage in *P. sojae* (Fahlgren et al., 2013). Diversity in the number of small RNA biogenesis genes has also been seen. *T. gondii* has a single homolog each of DCR, RDR, and AGO (Braun et al., 2010), whereas *Phytophthora* species have two DCR homologs with a diverse origin, a single RDR homolog, and a large and varied number of AGO homologs (Fahlgren et al., 2013; Bollmann et al., 2016). High-throughput sequencing in three *Phytophthora* species revealed two major size classes of small RNAs, 21-nt and 25/26-nt, which matches well with the existence of two DCR homologs (Vetukuri et al., 2011; Fahlgren et al., 2013).

Phylogenetic analyses of the DCR homologs in *Phytophthora* revealed a divergent evolutionary origin, with DCL1 clustering with plant and animal DCR-like homologs, and DCL2 clustering more basally in the tree with homologs of Drosha (Bollmann et al., 2016). Additionally, although both DCL1 and DCL2 have two RNase III domains, typical of DCR homologs, the other domains differ greatly. DCL1 is longer and includes the DEAD-box helicase and dsRNA binding domain, whereas DCL2 has a PAZ domain and a dsRNA binding motif. In plants and animals, for comparison, it is common for most if not all of these domains to be present in each homolog. Interestingly, *Phytophthora* species, similar to *Dictyostelium*, have a DEAD-box helicase and Helicase-C domains on the N-terminus of the RDR homolog. This possible domain transfer from the DCL2 homolog to RDR may suggest interaction of those proteins in the same pathway, lending support for DCL2 in the 25-26-nt sRNA pathway. The study also showed nuclear localization of both DCR homologs (Bollmann et al., 2016).

A recent study showed the first evidence for specific roles of AGO homologs in *Phytophthora* (Åsman et al., 2016). Phylogenetic analysis revealed that oomycete AGO homologs cluster with the AGO-like family and that the brown algae homolog is more closely related to oomycetes than to the diatom homologs. This study also confirmed that oomycetes have two well-defined AGO clades (Fahlgren et al., 2013), with AGO1 in a separate clade from the rest of the AGO homologs in the species tested, as well as differences in key residues in the PIWI domain (Åsman et al., 2016). Co-immunoprecipitation assays in *P. infestans* revealed that Ph_infeAGO1 primarily associates with 20-22-nt sRNAs, with a preference for 5′ C, Ph_infeAGO4 primarily associates with 24-26-nt sRNAs, with a strong preference for 5′ U, and Ph_infeAGO5 has no apparent size preference. In addition, sequencing the precipitated sRNAs revealed that Ph_infeAGO1 associates with the one confirmed miRNA (Fahlgren et al., 2013) and with sRNAs derived from Crinkler genes, protein-coding genes, and Gypsy long terminal repeat retrotransposons. In contrast, Ph_infeAGO4 associates with sRNAs derived from Helitron, Crypton, PiggyBac, and Copia transposons, and Ph_infeAGO5 associates with sRNAs derived from introns and intergenic regions (Åsman et al., 2016). In the *P. infestans* strain used for the study, there was an early indel in *Ph_infeAGO*3 which disrupted the protein and did not allow further testing. However, they showed that the N-terminus of Ph_infeAGO3 is Gly-Arg-rich, which in *T. brucei* and *T. gondii* has been shown to be important for AGO function (Shi et al., 2004; Musiyenko et al., 2012). Unlike the DCR homologs, Ph_infeAGO1 and Ph_infeAGO4 were shown to be localized to the cytoplasm (Åsman et al., 2016).

The goal of this study was to further refine the phylogenetic analysis of oomycete AGO homologs, both by deeper analysis of the broader eukaryotic tree, and a more in-depth analysis of a wider range of *Phytophthora* species. Unlike other Stramenopiles, *Phytophthora* species have diverse numbers of *AGO* homologs, and clade 7 species in particular show a conserved expansion of a tandem duplication common to *Phytophthora* species. Through real-time quantitative PCR (RT-qPCR), we documented dynamic transcript levels of *AGO* genes in *P. sojae*, in contrast to the steadier levels of transcript levels of the DCR and RDR homologs. We additionally document the syntenic relationships of the tandem duplication of *AGO* homologs and the presence/absence of key conserved residues in the MID and PIWI domains.

## Materials and Methods

### Selection of Genes for Phylogenetic Analyses

AGO and PIWI homolog sequences were initially identified using TBLASTN (NCBI) searches against species-specific genomic databases. Various queries were used, including full-length sequences or PAZ/PIWI domain sequences from oomycetes such as *Phytophthora sojae*. Species with partial or fully-sequenced genomes were selected from all available Chromalveolate groups, as well as species from other eukaryotic supergroups in order to assess ancient evolutionary relationships. A sample of AGO and PIWI homologs identified in previous phylogenetic analyses (Cerutti and Casas-Mollano, 2006; Yigit et al., 2006; Murphy et al., 2008) were included along with homologs from Chromalveolates that were previously less represented. The JGI database ^1^ was searched by querying specific organisms for the presence of annotated PAZ and PIWI domains. Oomycete homolog sequences were identified in the VBI Microbial Database ^2^ and the *P. infestans* database at the Broad Institute ^3^ (now at fungidb.org). Other genomes were made available by permission: *P. cinnamomi* (JGI; Wayne Reeve, pers. comm.) and *P. melonis* (Wenwu Ye, pers. comm.). Genomic sequences encompassing the identified Chromalveolate homologs were selected and coding sequences were manually identified and confirmed using Genscan (Burge and Karlin, 1997) or FGENESH ^4^. Protein domains were identified using Pfam database searches (Finn et al., 2010). To be retained in the analyses, an AGO homolog minimally had to contain a PAZ and a PIWI domain. An AGO-like homolog from the Archaea was also identified for use as an outgroup.

### Cloning and Sequencing of *P. sojae* *AGO* Homologs

*P. sojae* was used as the model *Phytophthora* species to validate *AGO* sequences. Total RNA was isolated from *P. sojae* strain P6497 mycelium (grown in cleared V8 broth) using TRIzol (Invitrogen, Carlsbad, CA, United States) from 250 μg total RNA with an Oligotex column (Qiagen, Germantown, MD, United States). cDNA was produced using the GeneRacer RACE Ready kit (Invitrogen) following the manufacturer’s guidelines, in order to capture the 5′ and 3′ ends of the mRNAs. Coding sequences from genomic database queries were predicted with Genscan (Burge and Karlin, 1997). Primers were designed using the Primer3 (Rozen and Skaletsky, 2000) plug-in in Geneious^5^ (Kearse et al., 2012). Primer sequences and locations are detailed in Supplementary Table 1 and Supplementary Figure 1. Genomic DNA isolated from mycelium with a FastPrep kit (MP Biomedicals, Solon, OH, United States) was used for initial primer testing to confirm the predicted genomic sequence and test the efficacy of the primers. All PCR products were amplified with Taq polymerase (Genscript, Piscataway, NJ, United States) following the manufacturer’s guidelines. All primer annealing temperatures were between 56–60°C and extension times were calculated using a rate of 1 kb per minute. As recommended by GeneRacer guidelines, amplification of the 5′ and 3′ UTRs utilized touchdown PCR, typically with a 1-min extension. Amplification of internal sequences typically involved a 60°C annealing temperature and 4-min extension. All PCR products were cloned with the TOPO-TA cloning kit (Invitrogen, Carlsbad, CA, United States). A minimum of two to three clones were sequenced for each product using the BigDye Terminator v. 3.1 Cycle Sequencing Kit on an ABI Prism 3730 Genetic Analyzer (Applied Biosystems, Carlsbad, CA, United States). The sequences were assembled and manually analyzed using Geneious software. *P. sojae* sequences have been deposited in GenBank under the following accession numbers: *PsAGO*1L (MH198153), *PsAGO*1S (MH198154), *PsAGO*2 (MH198155).

### RT-qPCR Analysis of Transcript Levels

Transcript levels of *AGO* genes were characterized in several life stages and in a time series of infected host tissue. Total RNA was isolated using TRIzol from *P. sojae* mycelium, zoospores, and germinated cysts (6 replicates each) or from two independent soybean infection experiments, each with three replicate hypocotyls collected at 3, 6, 12, 24, or 48 h-post-infection. Zoospores were produced by repeated washing of 11 day-old V8-200 plates of mycelium followed by overnight incubation at 14°C. Germinated cysts were produced by exposing zoospores to cleared V8 broth for 1 h. Soybean infection experiments followed the protocol of Qutob et al. (2000) in which infected hypocotyls were incubated at 28°C for 14 h in the light and 25°C for 10 h in the dark. 50 mg of TRIzol-isolated RNA from each replicate was processed through the AllPrep DNA/RNA Mini kit (Qiagen) following the manufacturer’s guidelines with the addition of one DNase I treatment. The Superscript III First-Strand Synthesis System for RT-PCR kit (Invitrogen, Carlsbad, CA, United States) was used to produce cDNA, followed with purification by phenol:chloroform extraction. cDNA was quantified with a Nanodrop ND-1000 (Thermo Scientific, Waltham, MA, United States) to allow for equal quantities of cDNA template in subsequent reactions. Primers were designed as described above. Standard real-time quantitative PCR reactions were performed with Fast SYBR Green Master Mix (Applied Biosystems, Carlsbad, CA, United States) on an ABI StepOnePlus Real-Time PCR System (Applied Biosystems, Carlsbad, CA, United States). Data was normalized to reference genes β-Tubulin (Ps109498) and WS41 (Ps137777; protein of the BAR-domain family) and analyzed with Bestkeeper (Pfaffl et al., 2004), REST (Pfaffl et al., 2002), and SAS (Cary, NC, United States) software.

### Phylogenetic Analyses

A phylogenetic analysis was conducted to establish the evolutionary history of *AGO* genes. Alignments of protein sequences (Supplementary Data Sheets 2, 3 and Supplementary Table 2) were created with MAFFT ^6^ (Kuraku et al., 2013; Katoh et al., 2017). The amino acid sequences encompassing the PAZ through PIWI domains were analyzed across a broad range of species, while all predicted domains were analyzed across the Stramenopiles. To reduce error introduced due to partial sequences and to minimize computational load, amino acid positions that were absent in a majority of the analyzed species were removed from the alignment (Baurain et al., 2010). Any protein sequences with significant gaps in the alignment were removed from the analysis. Species were identified in phylogenetic trees with an abbreviated name in the format “Ge_spec” (the first two letters of the generic name followed by the first four letters of the specific name). Gene number designations do not necessarily follow homology. Previous designations, either from the genomic or predicted transcript database or a previous publication (Cerutti and Casas-Mollano, 2006; Yigit et al., 2006; Murphy et al., 2008), were maintained; otherwise the number assignment was arbitrary when there was no available reference. *Phytophthora* gene number designations were changed to reflect the evolutionary relationships among homologs.

Condensed domain alignments for the broader phylogeny were used for Bayesian phylogenetic analyses using MrBayes-3.1.2 (Huelsenbeck and Ronquist, 2001; Ronquist and Huelsenbeck, 2003) with the following settings: mixed amino acid model of evolution with invgamma rates, unconstrained brlenspr, sample frequency of 50, 2 runs with 4 chains each, temperature of 0.2, and diagnostic frequency of 1,000. The phylogenetic analysis ran for 8 million generations with a final burn-in of 25%. The final consensus tree from the Bayesian analyses was obtained using maximum likelihood analysis using RAxMLPTHREADS-7.0.0 (Stamatakis, 2014); 1,000 bootstrap replications were performed. Chromalveolate alignments were analyzed in MEGA7 (Kumar et al., 2016) using the Neighbor-Joining method (Saitou and Nei, 1987) as their evolutionary distances were much smaller. The Poisson correction method (Zuckerkandl and Pauling, 1965) was used for estimating evolutionary distances, with removal of ambiguous positions for each sequence pair and a bootstrap test with 1,000 replicates (Felsenstein, 1985).

## Results

### Phylogenetic Analyses of the Chromalveolate AGO Homologs

**FIGURE 1:**
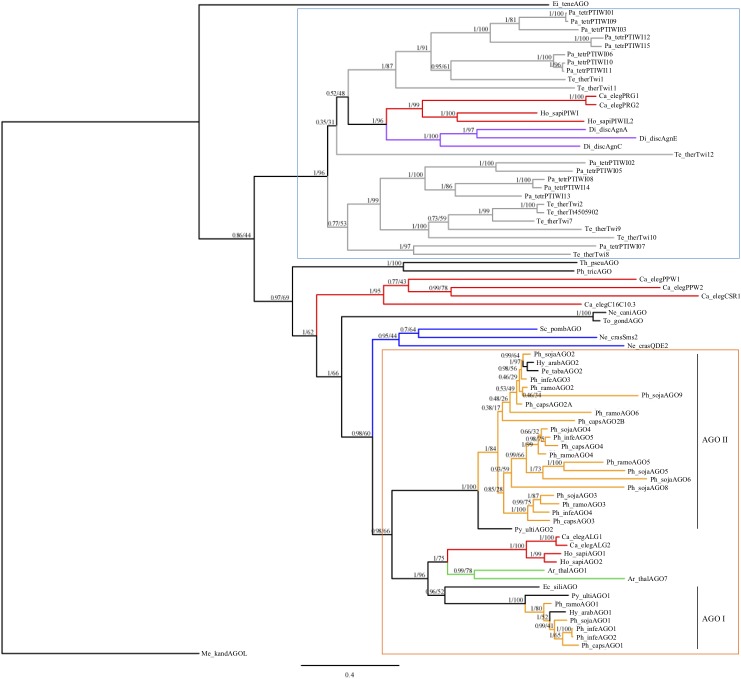
Consensus phylogram of AGO homologs based on PAZ through PIWI domains. Bayesian support and maximum likelihood values, respectively, are indicated. Branches are colored to denote major species groups: gray, ciliate; red, animal; purple, amoeba; blue, fungi; light orange, *Phytophthora*; green, plant. An archaea PIWI-like homolog (*Methanopyrus kandleri* AV19) was included as an outgroup (Shabalina and Koonin, 2008). Well supported PIWI-like (blue upper box) and AGO-like (orange lower box) clades are indicated, as well as oomycete AGO I and AGO II clades. Species are abbreviated as: Ge_spec (Supplementary Table 2).

To infer the evolutionary history of *Phytophthora* AGO homologs, we conducted a phylogenetic analysis of AGO homologs including species selected across the Chromalveolate supergroup and representatives of eukaryote AGO-like and PIWI-like homologs, including an Archaea PIWI-like homolog as an outgroup (Figure 1). The conserved PAZ through PIWI domains were aligned and used for Bayesian and maximum likelihood phylogenetic analysis. As expected, the ciliate AGO homologs formed a distinct group with PIWI-like homologs from *C. elegans*, humans, and *Dictyostelium*. Oomycete AGO homologs formed a separate group with AGO-like homologs from *C. elegans*, humans, and *Arabidopsis*. There were several groups that did not clearly cluster into either of these larger groups, namely homologs from diatoms, Apicomplexa, fungi and *C. elegans*. The Apicomplexan *Eimeria tenella* AGO and archaea PIWI-like homologs were both outgroups of the tree.

Within the oomycetes, the AGO-like sequences divided into two distinct clades (I and II). The AGO I clade contained the AGO1 homologs from *Phytophthora*, *Hyaloperonospora*, *Pythium*, and the single AGO homolog from *Ectocarpus siliculosis*; this clade grouped with the other eukaryotic AGO-like homologs. In clade I, most of the oomycete AGO1 sequences were present in single copy. The exception was *P. infestans*, which has a pair of recently duplicated genes, which encoded proteins that are 99.9% identical with a single amino acid difference. The AGO II clade contained exclusively oomycete sequences. This clade contained a single AGO2 homolog in two downy mildew species, including *Hyaloperonospora arabidopsidis*, *Peronospora tabacina*, as well as *Pythium ultimum*. However, there was evidence for multiple duplication and divergence events among the *Phytophthora* species resulting in sub-groups AGO2 to AGO9. Members of the AGO II clade in general have longer, undefined N-termini that are Gly-Arg-rich, whereas members of the AGO I clade have shorter, undefined N-termini that are Pro-rich.

### Annotation of Selected *P. sojae* AGO Homologs

To further investigate the structural differences between *Phytophthora* proteins in the AGO I and AGO II clades, we selected the *AGO* genes from *P. sojae* for detailed gene annotation. Nine *P. sojae* homologs of *AGO* were identified bioinformatically from the genome sequence v1.0 (VBI Microbial Database^2^), most with good support from RNAseq data at FungiDB ^7^. The original annotation of the reference genome did not predict nine homologs. *Ph_sojaAGO*5 and *Ph_sojaAGO*8 were bioinformatically annotated as one gene, and *Ph_sojaAGO*9 was not predicted at all, as these three homologs are very close together on the chromosome (discussed further below). Detailed bioinformatic analysis of the region allowed separation of the homologs into distinct predicted ORFs.

Of the nine AGO homologs identified, four were predicted to have all six of the conserved AGO domains (N-terminal, Linker 1, PAZ, Linker 2, Mid, and PIWI), and all except *Ph_soja*AGO8 displayed both PAZ and PIWI domains. The other five homologs, in which all six conserved domains were not predicted, still showed similar amino acid sequences across all predicted domain regions, albeit the conservation within the PIWI domain was the highest. Supplementary Figure 2 shows the predicted structures of *Ph_soja*AGO3 through *Ph_soja*AGO9.

**FIGURE 2:**
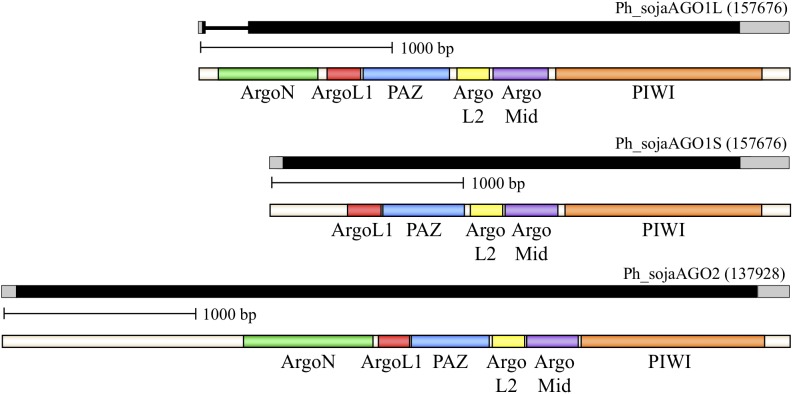
Organization of representative *P. sojae* Argonaute (AGO) homologs. Genes are labeled based on their Gene ID from the EuMicrobeDB database (eumicrobedb.org). In the genomic DNA diagrams, exons and introns are represented as black bars and lines, respectively. 5′ and 3′ UTRs are represented as gray bars. Conserved domains are indicated by colored bars in the mRNA diagrams: ArgoN, N-terminal Argonaute domain; ArgoL1, linker domain between N-terminus and PAZ domain; PAZ, PAZ domain named for the proteins Piwi Argonaute and Zwille; ArgoL2, linker domain between PAZ and PIWI lobes of Argonaute; ArgoMid, part of the PIWI lobe; PIWI, active domain for dsRNA-guided hydrolysis of mRNA.

We used sequence analysis of full-length cDNA clones to confirm predicted gene structures for three representative homologs: *Ph_sojaAGO*1 and *Ph_sojaAGO*2, representing the two major oomycete clades, and *Ph_sojaAGO*7 which was predicted to be a pseudogene and had a structure which was difficult to resolve. In the case of *Ph_sojaAGO*1, two different isoforms were found among the cDNA clones. One of these (*Ph_sojaAGO*1L), represented by 7 independent clones, displayed a more upstream start site as well as the removal of a 204 nt intron (Figure 2 and Supplementary Figure 3). The other isoform (*Ph_sojaAGO*1S), displayed a start site 351 bp downstream of the *Ph_sojaAGO*1L start site, with no intron, resulting in a predicted protein that is 64 residues shorter at the N-terminus than *Ph_sojaAGO*1L. Both *Ph_sojaAGO*1 and *Ph_sojaAGO*2 displayed typical UTRs: 5′ UTRs ranged from 10 to 64 bases in length, and 3’ UTRs ranged from 150 to 246 bases in length. The genes follow the general trends of *Phytophthora* genes, in which only one-third possess an intron (average length of 79 bases), and the average number of introns is 1.5 per gene (Kamoun, 2003; Tyler et al., 2006; Haas et al., 2009). Of the three annotated transcripts, the *Ph_sojaAGO*1L transcript contains an intron that is 204 bases in length, although the exon/intron boundaries are not the conventional GT..AG bases (Supplementary Figure 3). The GC content of the three transcripts are 62.6% (*Ph_sojaAGO*1L), 62.1% (*Ph_sojaAGO*1S), and 62.9% (*Ph_sojaAGO*2); typical of oomycete transcribed sequences (average of 58%). The sequences surrounding the transcription start site and translation start site also generally follow the trend seen in other *Phytophthora* genes (Supplementary Figure 3). Figure 2 displays annotated gene structures for *Ph_sojaAGO*1 and *Ph_sojaAGO*2, including the two alternative transcription start sites for *Ph_sojaAGO*1, along with conserved AGO domains in the encoded proteins.

*Ph_sojaAGO*7 was confirmed to be a pseudogene. The predicted *Ph_sojaAGO*7 coding sequence had no introns and had several premature termination codons. The AGO7 pseudogene transcript additionally had an unresolved start position. To allow for phylogenetic analyses, the best predicted transcript with the largest blocks of conserved domain sequences was used, which required many putative introns and had a GC content of 59.4%.

### Transcript Levels of *P. sojae* *AGO* Homologs

**FIGURE 3:**
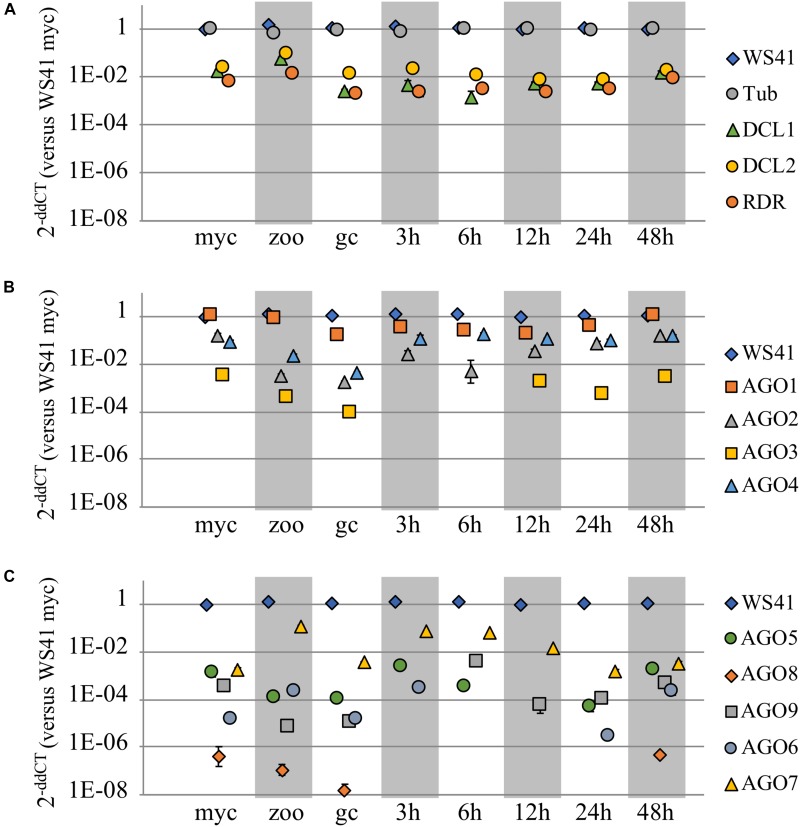
Transcript levels of *AGO* and other small RNA biogenesis genes. Three different life stages (myc, mycelium; zoo, zoospores; gc, germinated cysts) and an infection time series (3, 6, 12, 24, and 48 h post infection) were assayed in replicate by qRT-PCR to determine transcript levels of **(A)** *DCL*1, *DCL*2, *RDR*, and **(B,C)** the *AGO* homologs in *P. sojae* as compared to housekeeping genes b-tubulin and *WS41*. Each life stage or time series point is normalized to the housekeeping gene(s). Standard error bars are included, but in most cases the standard error bars are blocked by the symbol due to low value.

To investigate the respective functional roles of the *P. sojae* AGO homologs, qRT-PCR analysis was conducted to determine the transcript levels across three different life stages and across an infection time series. Housekeeping genes *WS41* and β-tubulin were used as internal controls (Supplementary Figure 4), and *DCL*1, *DCL*2, and *RDR* were also analyzed for comparison. The *DCL*1, *DCL*2, and *RDR* homologs showed fairly stable transcript levels across the stages and time series (Figure 3A and Supplementary Figure 5A), with a slight increase in levels in zoospores, as reported previously (Bollmann et al., 2016). The *AGO* homologs, in comparison, exhibited a wide range of transcript levels (Figures 3B,C and Supplementary Figures 5B,C). Whereas *AGO*1 had transcript levels nearly as high as the housekeeping gene *WS41*, *AGO*3 exhibited much lower levels, and in fact transcripts could not be detected at the 3 hours post infection (hpi) or 6 hpi treatments. *AGO*2, *AGO*4, and *AGO*7 exhibited similar transcript levels as *DCL*1, *DCL*2, and *RDR*, although only *AGO*7 showed increased levels in zoospores. *AGO*5, *AGO*6, *AGO*8, and *AGO*9, similar to *AGO*3, exhibited much lower transcript levels that were undetectable in some samples. Only *AGO*6 and *AGO*7 exhibited increased transcript levels in zoospores comparable to *DCL*1, *DCL*2, and *RDR*.

### Phylogenetic Analyses of the Oomycete AGO Homologs

**FIGURE 4:**
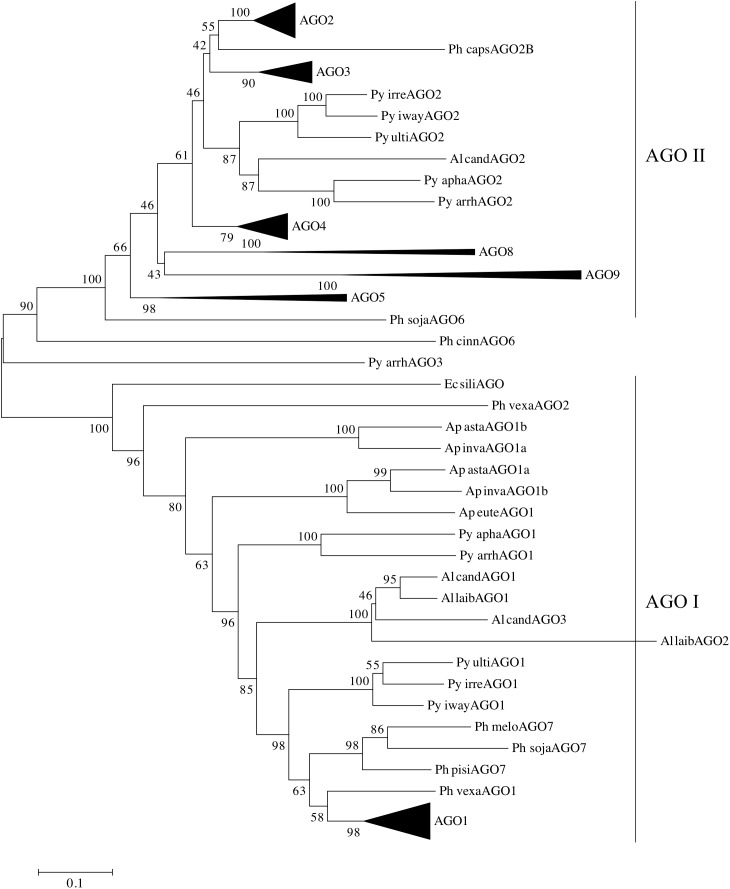
Phylogram of AGO homologs based on conserved domains. The neighbor-joining tree is based on evolutionary distances computed with the Poisson correction method using pairwise deletion of ambiguous sites. Bootstrap values (10,000 replicates) are indicated. Triangles denote collapsed ortholog groups as indicated; oomycete AGO I and AGO II clades are also noted. Species are abbreviated as: Ge spec (Supplementary Table 2).

In order to analyze the two clades of oomycete AGOs in more detail, the number of oomycete AGO homologs was expanded. AGO proteins were predicted bioinformatically from several newly sequenced *Phytophthora* genomes, as well as additional representative oomycete genomes from the genera *Pythium*, *Aphanomyces*, and *Albugo*. This set of homologs was less diverse than presented in Figure 1, and therefore a neighbor-joining tree was sufficient for phylogenetic analysis. The additional *Phytophthora* sequences revealed more details of the relationships between the different subclades. The results of the analysis are shown in Figure 4, where the major *Phytophthora* AGO groups are collapsed for clarity, while the full tree is shown in Supplementary Figure 6. As seen in Figure 1 and reported in previous studies (Fahlgren et al., 2013; Åsman et al., 2016), two major AGO clades are present in oomycetes: AGO I, comprised primarily of AGO1 and AGO7 homologs, and AGO II, comprised of most other AGO homologs. The two clades were defined as having 100% bootstrap support. AGO I contains representatives from all of the oomycete species examined, including representatives from the Saprolegniales (i.e., *Aphanomyces* species) and the single AGO homolog found in *E. siliculosus*. In the AGO II clade, representatives from the Pythiales (*Pythium* spp), Albuginales (i.e., *Albugo*) and downy mildews (*H. arabidopsidis*) were restricted to the AGO2-AGO3 cluster. The AGO4 to AGO9 homologs were restricted to representatives from *Phytophthora.* Within the different *Phytophthora* AGO subclades, the trees were largely congruent with the phylogenetic clades previously described for the genus (Blair et al., 2008), (color coded in Supplementary Figure 6).

*Pythium* and *Albugo* species both had two distinct groups of AGO homologs, one within AGO I, basal to the *Phytophthora* AGO1 sequences, and one within AGO II basal to the *Phytophthora* AGO2 and AGO3 groups. In contrast, the multiple AGO sequences within *Aphanomyces* all grouped together within AGO I, basal to the *Albugo* and *Pythium* sequences. Downy mildew (*Peronospora* and *Hyaloperonospora*) homologs of AGO1 and AGO2 grouped within the respective *Phytophthora* subclades within AGO I and AGO II.

The brown algae (*Ectocarpus siliculosus*) AGO homolog used as an outgroup lay basal to the AGO1 group, along with a few AGO sequences from *Aphanomyces*, *Pythium*, and *Phytopythium* species, which possibly may be encoded by pseudogenes. There was not enough sequence to resolve the position of *Phytophthora cinnamomi* AGO6 or *Pythium arrhenomanes* AGO3.

In *Phytophthora*, the AGO I clade consists primarily of single AGO1 homologs from each species. The exceptions are *P. infestans*, which has two nearly identical homologs, *P. pinifolia* with two homologs, and *P. cryptogea* with three homologs. Basal to the group of *Phytophthora* AGO1 sequences (Supplementary Figure 7) lie AGO1 homologs from *Hyaloperonospora arabidopsidis* and *Phytopythium vexans*, as well as a small group of *Phytophthora* AGO7 homologs (Figure 4). The remainder of the AGO I clade is made up of AGO1 homologs from *Pythium*, *Albugo* and *Aphanomyces* species.

The AGO II clade consists of multiple subgroups, each with multiple representatives from different *Phytophthora* species. Subgroups AGO2, AGO3, and AGO4 (Supplementary Figures 8–10) have representative genes from almost every *Phytophthora* clade present in the analysis. While not every species was represented, this may result from deficiencies in the genome assemblies used for identifying the AGO homologs. In most cases, there was a single representative of each subgroup in each species. Species from *Phytophthora* clades 3 and 4 appeared to be limited to AGO1 and AGO2 groups, although we cannot rule out the possibility that the sequence assembly had poor coverage over those regions. The AGO5/6/8/9 group, however, was primarily limited to *Phytophthora* clade 7 species, which exhibited a single homolog in each subgroup. The AGO5, 8, and 9 subgroups (Supplementary Figure 11) had strong support values. In contrast, the two AGO6 sequences, from *P. sojae* and *P. cinnamomi*, did not group together strongly and may not have a recent common origin.

### Genomic Location of a Subset of Oomycete AGO Homologs

**FIGURE 5:**
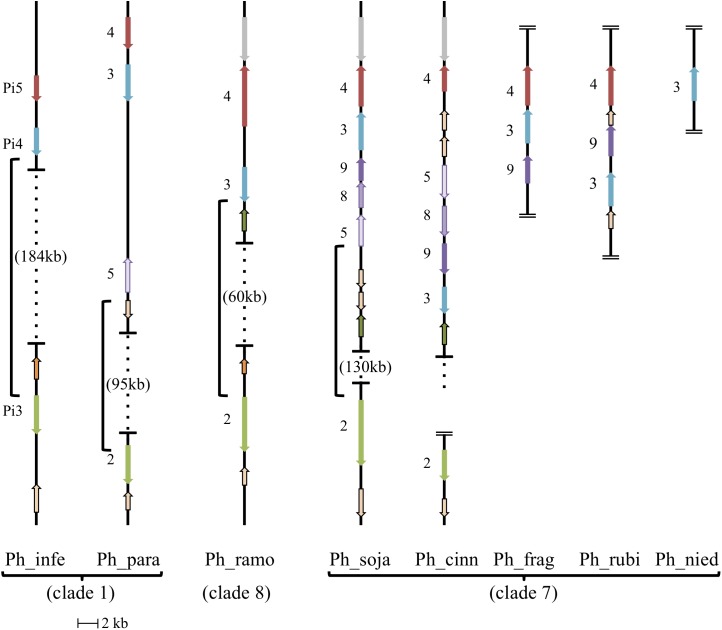
Synteny of AGO II clade orthologs. Diagram showing relative positions and orientations of AGO II clade orthologs which lie on the same contig. Horizontal lines with dotted vertical lines in between, have the genomic span between the closest orthologs indicated with braces (open ended dotted line indicates the contig continues, but does not have any further relevant orthologs). Double horizontal lines at the end of a contig indicate the end of the contig, where assembled contigs were too short to include all listed orthologs, in which case placement is hypothesized from related species. All AGO II clade orthologs are indicated with their gene number, and color coordinated to indicate orthology/synteny. Other conserved, syntenic genes surrounding the AGO II clade orthologs are noted: Glycosyl hydrolase family (tan with black border), Alpha/beta hydrolase fold family (green with black border), ER lumen protein retaining receptor family (orange with black border), and unknown protein (syntenic; gray). *Phytophthora melonis* (*Ph_melo*) has the same gene order and orientation as *Phytophthora sojae* (*Ph_soja*), although a homolog for *AGO*2 was not identified. *Phytophthora pisi* (*Ph_pisi*) likely has the same gene order as *Phytophthora sojae*, although the contig with *AGO*4-3-9-8-5 is cut off before the *AGO*5 homolog, which is missing in gene predictions, and *AGO*2 is on a different, shorter contig. *Phytophthora ramorum* (*Ph_ramo*) also has an *AGO*5 homolog, however, it is on a different contig.

Evolutionary relationships among the AGO homologs were further investigated by looking for evidence of synteny within homolog clusters. As shown previously (Fahlgren et al., 2013) in *P. sojae*, *P. infestans*, and *P. ramorum*, *AGO*2 lies on the same contig as *AGO*3 and *AGO*4 (the corresponding *P. infestans* genes are named *PiAGO*3, *PiAGO*4 and *PiAGO*5, respectively). As shown in Figure 5, our analysis revealed that *AGO*5, *AGO*8, and *AGO*9 are also within this gene cluster in the case of *P. sojae*. We then analyzed a wider set of *Phytophthora* genome sequences to look for syntenic *AGO* gene clusters (Figure 5). In *P. infestans* and *P. ramorum*, the *AGO*4, *AGO*3, and *AGO*2 homologs lie in the same order on the same contig, although the spacing between *AGO*4 and *AGO*3 homologs is small (3 kb, 4 kb) and the spacing between *AGO*3 and *AGO*2 homologs is large (187 kb, 60 kb). All *P. infestans* homologs lie in the same orientation, whereas in *P. ramorum* *AGO*4 lies in the opposite orientation of *AGO*3 and *AGO*2. In comparison, *P. sojae* is more complex and has *AGO*4, *AGO*3, *AGO*9, *AGO*8, *AGO*5, and *AGO*2 in that order. Again, *AGO*2 is separated from the other homologs by a large distance (130 kb), whereas the total span from the predicted start codon of *AGO*5 to the predicted stop codon of *AGO*4 is small (18 kb). The cluster of homologs from *AGO*5 to *AGO*4 all lie in the opposite orientation to *AGO*2 in *P. sojae*. *Phytophthora* species in clade 7 dominate the *AGO*5/8/9 group of homologs. *P. melonis* and likely *P. pisi* (truncated contig) show a similar gene order and orientation as *P. sojae*. *P. cinnamomi* also has all five tandem homologs in the same orientation, but in a different order than *P. sojae*: *AGO*4, *AGO*5, *AGO*8, *AGO*9, and *AGO*3. *P. fragariae* and *P. rubi* (Tabima et al., 2017) have three of the five clustered homologs (*AGO*4, *AGO*3, *AGO*9) in the same orientation, but different orders, and only *AGO*3 of the tandem set was identified in *P. niederhauserii*; however, we cannot rule out the possibility that sequences were missed in the genome assembly. Only two other genes grouped within the AGO5/8/9 group of homologs, specifically the AGO5 group, but these genes were not physically as close to the *AGO*4 and *AGO*3 genes as in clade 7 species. In *P. parasitica*, the distance between *AGO*5 and *AGO*3 is larger than in any other species, and in *P. ramorum* the *AGO*5 gene is on another contig. Surrounding syntenic genes can indicate boundaries of large changes between species, for example the unique inversion of multiple tandemly arranged genes between *P. sojae* and *P. cinnamomi* (*AGO*3,9,8,5; glycosyl hydrolases).

### Analysis of Predicted Key Residues

**FIGURE 6:**
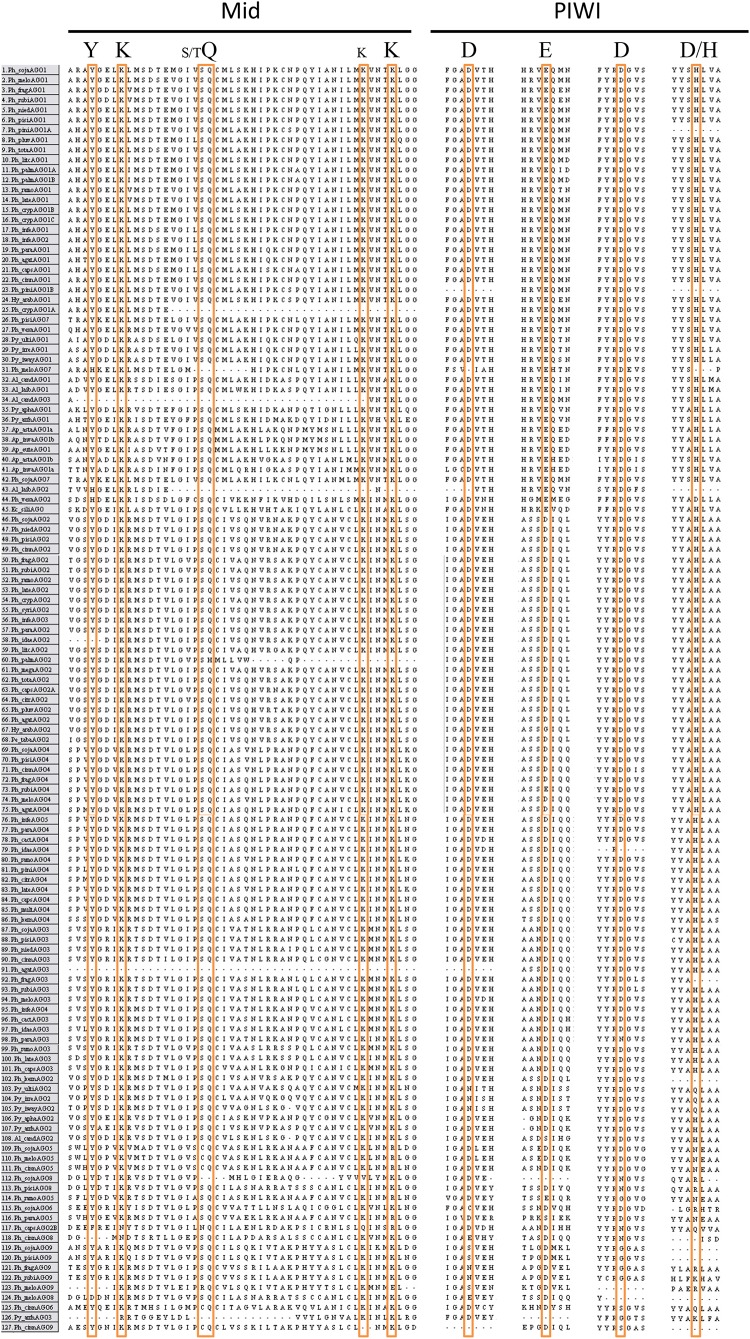
Alignment of key conserved residues in the Mid and PIWI domains of Chromalveolate AGO homologs. Amino acid sequence regions from MAFFT alignment of Chromalveolate AGO homologs with key residues highlighted. Conserved Mid domain residues: YKQK (S/T before the Q and an extra K before the final K have also been shown to have significance in some cases); conserved PIWI domain residues: DEDD/H. Species names as in Figure 4.

To assess whether the AGO homologs had the required amino acids for RNA cleavage activity, positions of known conserved residues in the MID and PIWI domains were compared (Figure 6). In the MID domain, the amino acids Y-K-(S/T)Q-(K)K are critical for binding of the 5′ phosphate of the guide sRNA, while other residues in the MID domain can confer specificity of nucleotide binding in some species (Boland et al., 2010; Frank et al., 2010). In the majority of the AGO proteins in our analysis, these residues are perfectly conserved. The exceptions, namely *Ph_crypAGO*1a, *Ph_meloAGO*7, *Al_laibAGO*2, *Al_candAGO*3, *Py_vexaAGO*2, *Py_arrhAGO*3, *Ph_idaeAGO*2, *Ph_agatAGO*3, *Ph_capsAGO*2B, *Ph_sojaAGO*8, *Ph_cinnAGO*8, *Ph_meloAGO*8, *Ph_cinnAGO*9, and *Ph_meloAGO*9, may result from incomplete sequences or the predicted proteins potentially being pseudogenes. The AGO5 group all have the residues Y-K-(S/C)Q-(K)R, which may or may not alter function. In the PIWI domain, the amino acids D-E-D-D/H are required, but not sufficient for cleavage of the target mRNA (slicer activity) (Nakanishi et al., 2012; Sheng et al., 2014). In agreement with the report by Åsman et al. (2016), the AGO I clade proteins from our analysis primarily contained the D-E-D-H residue pattern, while the majority of the AGO II clade proteins contained a D-D-D-H residue pattern, which may or may not affect the RNA cleavage ability of the protein. As with the MID domain residues, many of the *Phytophthora* clade 7 tandem repeat homologs showed variation in the conserved residues, including the *AGO*5 group (D-D-D-N, D-E-G-N, D-S-D-N), *Ph_sojaAGO*6 (C-D-D-R), *Ph_cinnAGO*6 (D-D-S-Q), the *AGO*8 group (D-_-N-R, D-D-N-R, E-D-N-H, E-D-_-_), and the *AGO*9 group (S-D-G-_, N-D-G-R, N-D-G-K, S-D-_-R, E-D-S-R). Additional changes include *AGO*2 from *H. arabidopsidis* (D-G-D-H), *AGO*2B from *P. capsici* (D-D-N-Q), *AGO*4 from *P. rubi* (D-E-D-H), *AGO*3 from *P. parasitica* (D-D-N-H), and *AGO*2 from several *Pythium* species (N-D-D-Q).

## Discussion

Our analysis has provided a refinement in our understanding of the evolutionary history of oomycete AGO, in particular for the AGO II clade. Our results support the hypothesis that there was one ancestral AGO2 gene followed by formation of paralogs. The AGO I and AGO II clades likely diverged early in oomycete evolution as they are both present in *Phytophthora*, *Peronospora*, *Pythium*, and *Albugo* species, but not in *Aphanomyces* or brown algae. The more extensive duplication and divergence of genes within the AGO II clade appears however, to be restricted to the genus *Phytophthora*. The presence of *AGO*2, *AGO*3, and *AGO*4 on the same genome sequence contig (where the contigs are sufficiently) long further supports this idea. *Phytophthora* clade 7 in particular shows even more extensive duplication and divergence where expansion of *AGO*3 and *AGO*4 has produced tandem arrays of five *AGO* homologs, with rearrangements of the order and orientation of the genes among species. Gene duplications exist in some of the other *Phytophthora* species, such as the recent duplication of *AGO*1 in *P. infestans*, but currently only in clade 7 is there evidence for a clade-specific, tandemly arrayed gene expansion, although we cannot rule out this phenomenon may be present in other species we did not include.

The different *P. sojae* *AGO* genes exhibit considerable variation in gene structure. *P. sojae AGO*1, *AGO*2, *AGO*3, and *AGO*4 consist of only one exon that encodes all of the predicted conserved AGO domains, whereas *AGO*5 has two predicted exons and is not predicted to have the Mid domain, and *AGO*6 has three predicted exons and lacks conserved sequences matching the N-terminal and Mid domains. *Ph_sojaAGO*9, with five predicted exons, lacks conserved sequence for the Mid domain, and *Ph_sojaAGO*8 with six predicted exons is only predicted to have the N-terminal, Linker 2, and PIWI domains. We cannot rule out that some gene model predictions are incorrect for those *AGO* genes which were not cloned, but the RNAseq data that is available supports the predictions, and in most cases the predicted introns are short. *Ph_sojaAGO*7 likely is a pseudogene, even though it exhibits reasonably high transcript levels compared with the other *AGO* genes. When cDNA was cloned for *Ph_sojaAGO*7, there were inconsistent results for the 5′ end of the gene, with each different reading frame represented by multiple clones and variation in initial short introns. Other clones further in the sequence of the gene revealed no other introns, even though each reading frame has many stop codons. Predictions of each reading frame showed representation of all but the N-terminal domain, but the conserved domains were scattered across the different frames. The best computationally predicted gene structure, which included Linker 1, PAZ, and PIWI domains, was used for the phylogenetic analysis. Where conserved domain predictions were absent in *P. sojae* AGO homologs, it is likely the matching scores of the residues were simply too low to predict the corresponding Pfam model, as the amino acid alignments showed conservation throughout the majority of the sequences. This divergence from the consensus could also be seen in the conservation of key AGO residues required for function. All of the AGO I clade proteins showed conservation of key residues in both the MID and PIWI domains. In contrast, in the AGO II clade, the MID domain residues were better conserved than the PIWI domain residues, especially among proteins encoded by the clade 7 tandem gene clusters. It is unknown if the variation in conserved residues in clade II proteins could be a reflection of differences in function, for example use of translation repression rather than RNA cleavage, or if many of the genes in the expanded clusters of the clade are pseudogenes. We also cannot rule out the possibility that some predicted gene models might be incorrect, leading to misalignment of key residues.

The differences in transcript levels and patterns of the various *AGO* genes in *P. sojae* further highlight the divergences in potential function among the genes, though we note that transcript levels do not always accurately reflect protein levels. Whereas the *DCL1*, *DCL2*, and *RDR* genes in *P. sojae* exhibit fairly stable transcript levels, comparable to housekeeping genes, the *AGO* genes exhibited very different transcript levels. *AGO*1 exhibited constitutive transcript levels across tissue types nearly as high as the housekeeping genes tested and well above *DCL1*, *DCL2*, and *RDR*. *AGO*2 and *AGO*4 also exhibited quite consistent transcript levels, except for some reduction in zoospores and germinating cysts, and at a level below *AGO*1 and above *DCL1*, *DCL2*, and *RDR*. *AGO*3 and *AGO*5 exhibited levels around 10-fold lower than *AGO*2 and *AGO*4, while *AGO*6 and *AGO*9 transcripts levels were 100-fold lower and *AGO*8 levels were 1000-fold lower. In some samples these weakly transcribed *AGO* genes exhibited no detectable transcripts. The large variation in transcript levels among the different *AGO* genes also suggests distinct differences in function and/or that some are pseudogenes. The large variation in transcript levels among *AGO*3 through *AGO*9 occurred despite the colocalization of the genes in the genome. Surprisingly, transcript levels from the pseudogene *AGO*7 were almost as high as those from *AGO*2 and *AGO*4. None of the *AGO* genes showed strong tissue-specific transcript levels, except that *AGO*2, *AGO*3, *AGO*4, and *AGO*9 transcript levels showed some reduction in zoospores and germinating cysts. In summary, at least one AGO clade I gene and one *AGO* II gene always had transcript levels comparable to housekeeping genes but some AGO paralogs show substantial variation.

As indicated previously (Fahlgren et al., 2013; Jia et al., 2017), the presence of two size classes of small RNA (sRNA) in *Phytophthora* species correlates with the two major AGO clades. Åsman et al. (2016) demonstrated a physical association between the *P. infestans* PiAGO1 protein and 20-22 nt sRNAs and a miRNA, and between the AGO3 homolog PiAGO4 and 24-26 nt sRNAs. Interestingly, they found that the *AGO*2 homolog (named *PiAGO*3) had an early indel and a truncation in their strain of *P. infestans*, making it apparently non-functional. Their findings suggest that in *Phytophthora*, AGO I clade proteins may be specific to the smaller size class of sRNAs, and AGO II clade proteins may be specific to the larger sRNA size class. We and others (Fahlgren et al., 2013; Åsman et al., 2016; Jia et al., 2017) previously found a smaller size class of sRNAs that were derived from *Crinkler* and RXLR effector genes that are important for *Phytophthora* infection of the host plant; this is the class associated with PiAGO1 (Åsman et al., 2016). Only one miRNA has been identified in *Phytophthora* (Fahlgren et al., 2013), but if the AGO I proteins function in all 20-22 nt size sRNA RISC complexes, and not just miRNA pathways, then the high transcript levels of *AGO1* seen in *P. sojae* may be important for other types of small RNA regulation. The transcript level of AGO1 is in fact high in both mycelium and zoospores, but drops six-fold in germinated cysts and remains at levels three to five-fold below the mycelial level until 48 hpi, similar to the pattern seen for DCL1, DCL2, and RDR. Perhaps AGO1 regulation of effector transcript levels is relaxed upon encounter with a host plant. Further analysis of AGO homologs and their partner small RNAs will further reveal how *Phytophthora* species use small RNAs to regulate their own transcript levels and their interaction with host plants.

## Author Contributions

All authors contributed to designing and analyzing experiments, specifically SB was the primary contributor with help from CP and NG on design of transcript level assays and NG and BT on phylogenetic analysis. All authors also contributed to the drafting, revising, and final approval of the manuscript and are accountable for its accuracy.

## Conflict of Interest Statement

The authors declare that the research was conducted in the absence of any commercial or financial relationships that could be construed as a potential conflict of interest.

## Footnotes

1 genome.jgi-psf.org

2 eumicrobedb.org

3 www.broadinstitute.org/annotation/genome/phytophthora_infestans/MultiHome.html

4 linux1.softberry.com/berry.phtml

5 http://www.geneious.com

6 mafft.cbrc.jp/alignment/server

7 fungidb.org
