# Supplementary material for: Expansion and Divergence of Argonaute Genes in the Oomycete Genus Phytophthora
Source: Front Microbiol. 2018 Nov 30;9:2841. doi: 10.3389/fmicb.2018.02841 (PMC6284064; doi:10.3389/fmicb.2018.02841)
Supplement: Supplementary file 1 [file Table_1.DOCX]

Supplementary Table 1. Primers used for cloning and sequencing

| Forward Primer Name | Forward Primer Sequence | Reverse Primer Name | Reverse Primer Sequence | Target Gene | Gene position cloned |
| --- | --- | --- | --- | --- | --- |
| 5'RACE forward | CGACTGGAGCACGAGGACACTGA | AGO1-5 | GGGTCCGCCTCCTTCACCACCGCCGA | AGO1 | TSS-644 |
| 5'RACE nested forward | GGACACTGACATGGACTGAAGGAGTA | AGO1-5n | GCGCCTTGGGCGGGCCATCGTTGCC | AGO1 | TSS-445 |
| AGO1-front | AGCTTCGGCAACGATGGCCC | AGO1-end | GCATATCCCCGACTCCACAA | AGO1 | 415-2451 |
| AGO1-1082F | AGGACAGCGCGGAGAACA | used for sequencing primer | | AGO1 | (1082…) |
| AGO1-3 | CCGACGTGACGCACCCGAGCCCGAT | 3'RACE reverse | GCTGTCAACGATACGCTACGTAACG | AGO1 | 2018-term |
| AGO1-3n | CGATTCGGGCGCAAGGCCACCGCGT | 3'RACE nested reverse | CGCTACGTAACGGCATGACAGTG | AGO1 | 2114-term |
| 5'RACE forward | CGACTGGAGCACGAGGACACTGA | AGO2-5 | TAGTCGCCCCCGCCGCGCTGGTCGT | AGO2 | TSS-197 |
| 5'RACE nested forward | GGACACTGACATGGACTGAAGGAGTA | AGO2-5n | CGCCATACCCTCCGCCGCCGCTGCC | AGO2 | TSS-109 |
| AGO2-front | GCTACGACCAGCGCCGCTAC | AGO2-end | CCGTGGATGCACCGTCCGAG | AGO2 | 44-3784 |
| AGO2-front | GCTACGACCAGCGCCGCTAC | AGO2-393R | GCCTCGGTCGTCGTATCCAG | AGO2 | 44-393 |
| AGO2-276F | GCGTGGCTATGAAGGAAGTC | AGO2-2917R | TGCGCTTAATGTCACCGTAG | AGO2 | 276-2917 |
| AGO2-895F | CGTGGAGGGGACCGAGGAGG | used for sequencing primer | | AGO2 | (895…) |
| AGO2-2819F | GCTTCAAGGAGTTGGAGTCG | AGO2-end | CCGTGGATGCACCGTCCGAG | AGO2 | 2819-3784 |
| AGO2-3 | CTCGGGCATGGGGTCGCGGCCGTCGA | 3'RACE reverse | GCTGTCAACGATACGCTACGTAACG | AGO2 | 3117-term |
| AGO2-3n | CATTTGGCTGCGGGTCGCGCGCGCTT | 3'RACE nested reverse | CGCTACGTAACGGCATGACAGTG | AGO2 | 3724-term |
| 5'RACE forward | CGACTGGAGCACGAGGACACTGA | AGO7-5-2 | CGCTCTTCCGGCCGCTTGCGCTCAT | AGO7 | TSS-527 |
| 5'RACE forward | CGACTGGAGCACGAGGACACTGA | AGO7-5-3 | GCTGCGTCCCGTCGGCTCCGCTTCA | AGO7 | TSS-904 |
| AGO7-front | CGCAGAACCGCCTCGTGGTC | AGO7-end | CGGACGGCACCATCGACACC | AGO7 | 76-1812 |
| AGO7-3-3 | GCGCCACAGCACGCGCCTGTTTCCT | 3'RACE reverse | GCTGTCAACGATACGCTACGTAACG | AGO7 | 1561-term |
