# Supplementary material for: Expansion and Divergence of Argonaute Genes in the Oomycete Genus Phytophthora
Source: Front Microbiol. 2018 Nov 30;9:2841. doi: 10.3389/fmicb.2018.02841 (PMC6284064; doi:10.3389/fmicb.2018.02841)
Supplement: Supplementary file 3 [file Data_Sheet_1.pdf]

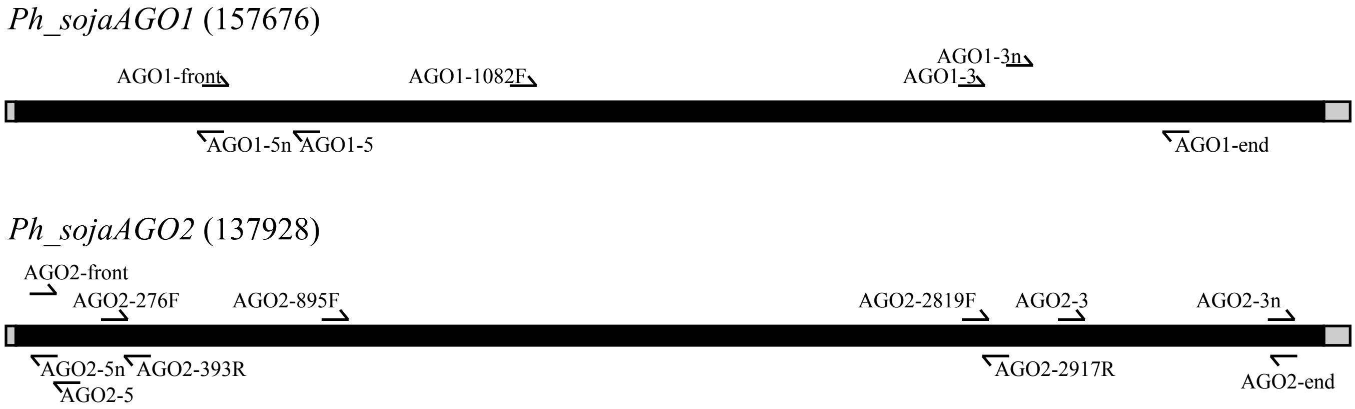

Supplementary Figure 1. Primers for cloning *P. sojae* AGO1 and AGO2. Genes are labeled with their Gene ID from the Eumicrobe Database. The coding sequence is represented as black bars, and the UTRs are represented as grey bars. Positions of primers are as detailed in Supplementary Table 1.

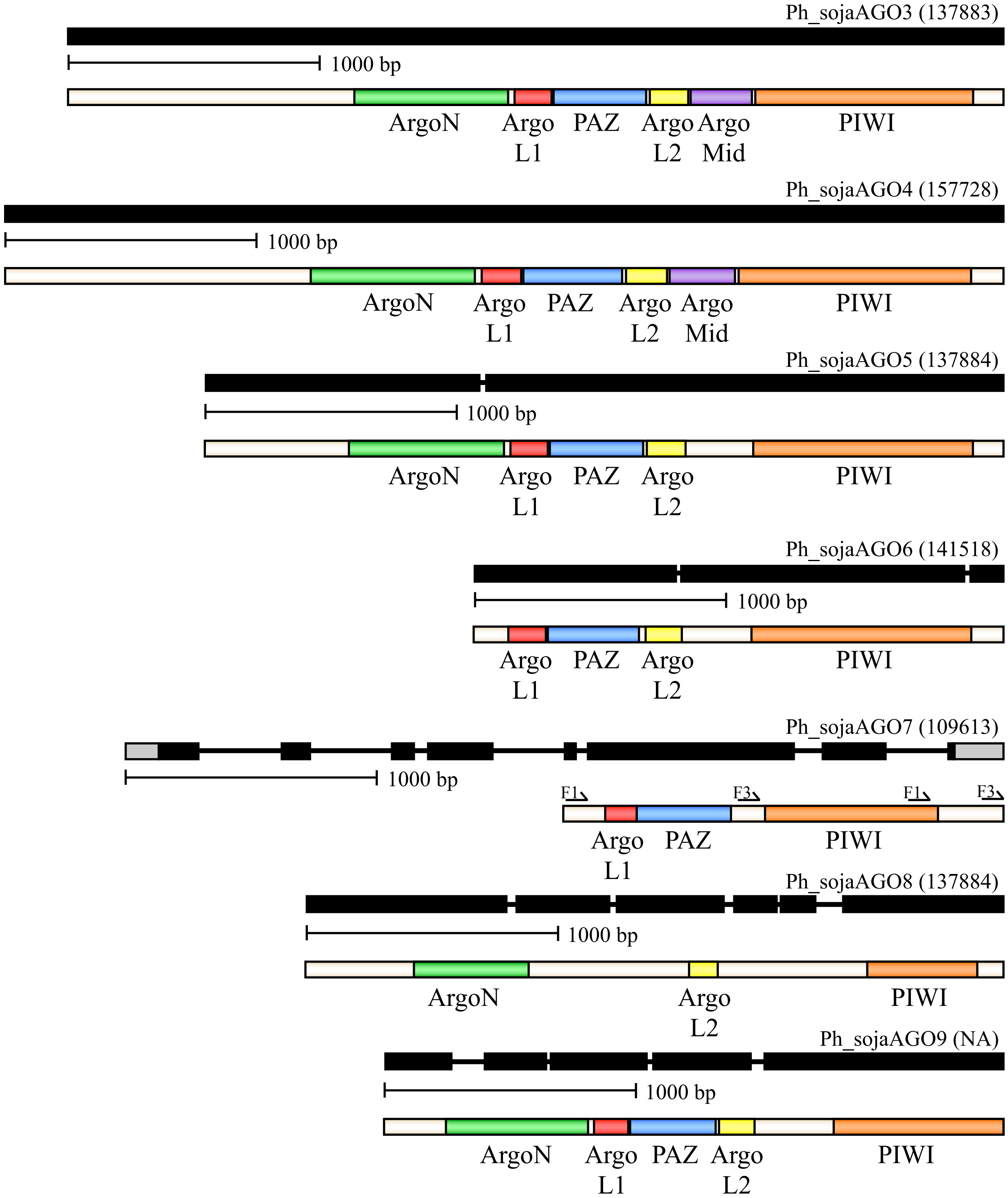

Supplementary Figure 2. Organization of predicted *P. sojae* Argonaute (AGO) homologs. Genes are labeled followed by their Gene ID (if predicted) from the EuMicrobeDB Database (eumicrobedb.org). In the genomic DNA diagrams, exons and introns are represented as black bars and lines, respectively. Representative 5′ and 3′ UTRs for AGO7 are represented as grey bars, with best predicted exon/intron boundaries for alignment. The corrected pseudogene sequence for AGO7 required three frameshifts (reading frame indicated with arrows on mRNA structure) to include the maximal amount of AGO conserved domain sequence. Conserved domains are indicated by colored bars in the mRNA diagrams: ArgoN, N-terminal Argonaute domain; ArgoL1, linker domain between N-terminus and PAZ domain; PAZ, PAZ domain named for the proteins Piwi Argonaute and Zwille; ArgoL2, linker domain between PAZ and PIWI lobes of Argonaute; ArgoMid, part of the PIWI lobe; PIWI, active domain for dsRNA-guided hydrolysis of mRNA.

A. Transcription start site

GCTCATTYBNNNWTTY – consensus
ATTCATTCCGCAAGAT – AGO1L 10b 5′ UTR
CAACCACTTCAAGGTC – AGO1S 55b 5′ UTR
TGTCAGTCCTCAACTC – AGO2 64b 5′ UTR
CTTCATTCAACAGATA – AGO7 88b 5′ UTR (2 clones)
ACTCGACTGGCAGAAG – AGO7 131b 5′ UTR (2 clones)
TTTCAGTTGCAAAATA – AGO7 147b 5′ UTR (1 clone)
TGGCAGAAGGGCGAGC – AGO7 124b 5′ UTR (1 clone)

B. Translation start site

ACCATGA – consensus
AAGATGC – AGO1L
AGCATGG – AGO1S
GCCATGA – AGO2

C. Exon/intron boundaries

GTRNGT...YAG – consensus
GCGAGC...CGC – AGO1L intron (204b)

Supplementary Figure 3. Gene structure consensus sequence comparisons of *P. sojae* AGO1, AGO2, and AGO7. Transcription start site (A), translation start site (B), and exon/intron boundary (C) sequences for AGO1, AGO2, and AGO7 (A only) are compared against the *Phytophthora* consensus sequence as described by Kamoun (2003). Underlined bases indicate: (A) first base of cDNA, (B) translation start codon, and (C) first and last bases of intron.

(A)

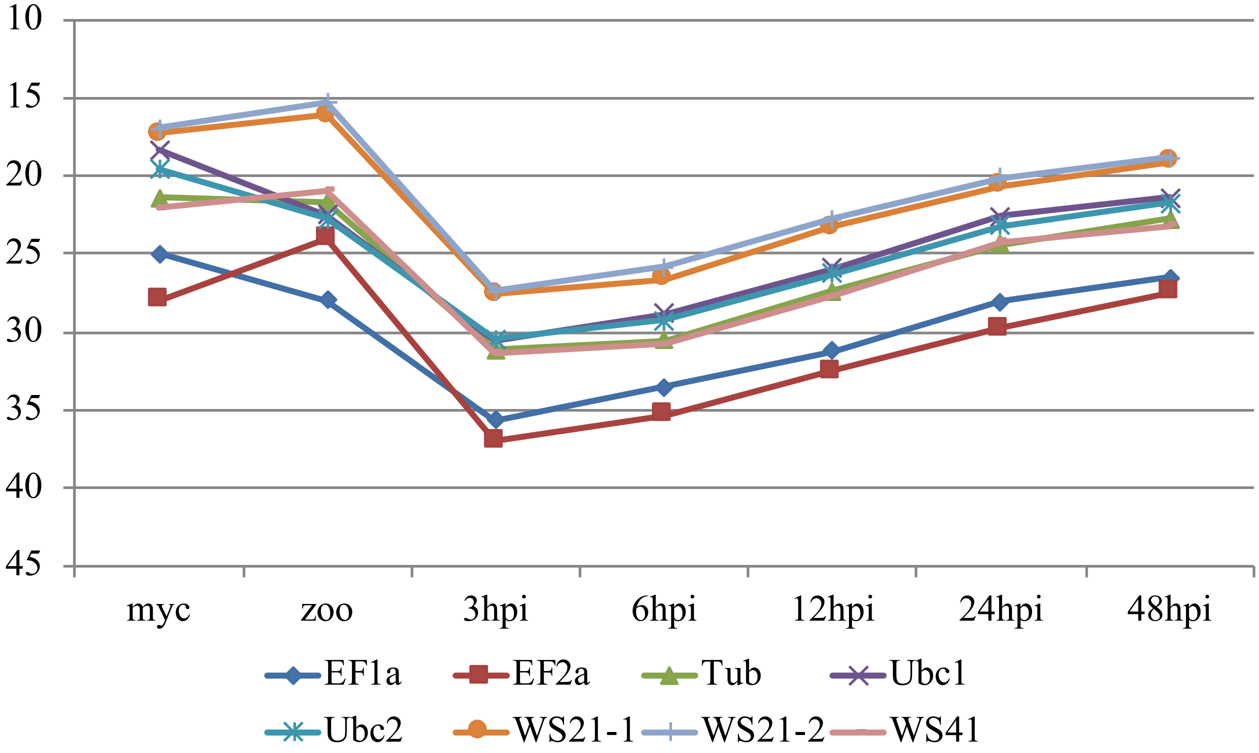

(B)

Gene name	Stability value	Standard error
Tub	0.011	0.015
WS41	0.014	0.013
Ubc2	0.047	0.016
WS21-1	0.056	0.018
EF2a	0.057	0.018
EF1a	0.061	0.019
Ubc1	0.062	0.020
WS21-2	0.069	0.021

(C)

Gene name	Stability value	Standard error
WS41	0.019	0.013
Tub	0.032	0.013
DCL2	0.042	0.014
Ubc2	0.057	0.016
RDR	0.061	0.017
DCL1	0.074	0.020

Supplementary Figure 4. Selection of housekeeping genes for RT-qPCR analysis. (A) Two different life stages (myc: mycelium, zoo: zoospores) and an infection time series (3hpi, 6hpi, 12hpi, 24hpi, 48hpi; hpi: hours post infection) were assayed to determine suitability of selected housekeeping genes for use in the RT-qPCR analysis of transcript levels in P. sojae. (B) Ranked summary of Normfinder results. Lower stability values (estimated transcript level variation within experimental sample set) indicate more consistent transcript levels versus the other genes. (C) Ranked summary of Normfinder results. DCL1, DCL2, and RDR were compared more extensively (three replicates plus an additional life stage – germinated cysts) to the top three housekeeping genes from the initial comparison (b-tubulin, WS41, and Ubiquitin conjugating enzyme 2).

(A)

	WS41	Tub	DCL1	DCL2	RDR
Mycelium	1.00<br>+/- 0.10	0.96<br>+/- 0.10	1.6 E-02<br>+/- 0.34 E-02, B	2.2 E-02<br>+/- 0.33 E-02, B	5.8 E-03<br>+/- 0.85 E-03, B/C
Zoospores	1.48<br>+/- 0.12	0.64<br>+/- 0.054	5.9 E-02<br>+/- 0.69 E-02, A	8.6 E-02<br>+/- 0.89 E-02, A	1.3 E-02<br>+/- 0.19 E-02, A
Germinated cysts	1.15<br>+/- 0.099	0.83<br>+/- 0.071	2.7 E-03<br>+/- 0.39 E-03, C	1.2 E-02<br>+/- 0.16 E-02, B	2.0 E-03<br>+/- 0.30 E-03, C
3 hpi	1.29<br>+/- 0.13	0.74<br>+/- 0.074	4.3 E-03<br>+/- 2.3 E-03, B/C	1.9 E-02<br>+/- 0.33 E-02, B	2.0 E-03<br>+/- 0.58 E-03, B/C
6 hpi	1.07<br>+/- 0.094	0.90<br>+/- 0.079	1.5 E-03<br>+/- 0.84 E-03, B/C	1.2 E-02<br>+/- 0.34 E-02, B	2.8 E-03<br>+/- 0.75 E-03, B/C
12 hpi	1.02<br>+/- 0.093	0.94<br>+/- 0.085	5.6 E-03<br>+/- 0.59 E-03, B/C	6.9 E-03<br>+/- 1.1 E-03, B	2.3 E-03<br>+/- 0.38 E-03, C
24 hpi	1.19<br>+/- 0.10	0.81<br>+/- 0.071	5.6 E-03<br>+/- 0.57 E-03, B/C	6.8 E-03<br>+/- 0.79 E-03, B	3.1 E-03<br>+/- 0.32 E-03, C
48 hpi	0.99<br>+/- 0.085	0.97<br>+/- 0.083	1.6 E-02<br>+/- 0.23 E-02, B/C	1.6 E-02<br>+/- 0.23 E-02, B	8.3 E-03<br>+/- 1.3 E-03, A/B

(B)

	WS41	AGO1	AGO2	AGO3	AGO4
Mycelium	1.00<br>+/- 0.11	1.02<br>+/- 0.17, A	1.7 E-01<br>+/- 0.29 E-01, A	3.2 E-03<br>+/- 0.55 E-03, A	8.9 E-02<br>+/- 1.3 E-02, A/B
Zoospores	1.36<br>+/- 0.17	0.80<br>+/- 0.13, B	3.1 E-03<br>+/- 0.70 E-03, C	4.0 E-04<br>+/- 0.76 E-04, B	2.2 E-02<br>+/- 0.40 E-02, B
Germinated cysts	1.13<br>+/- 0.11	0.16<br>+/- 0.020, C	1.7 E-03<br>+/- 0.24 E_03, C	8.8 E-05<br>+/- 1.2 E-05, B	4.2 E-03<br>+/- 0.55 E-03, B
3 hpi	1.19<br>+/- 0.13	0.32<br>+/- 0.041, B/C	2.7 E-02<br>+/- 0.88 E-02, B/C		1.2 E-01<br>+/- 0.42 E-01, A
6 hpi	1.22<br>+/- 0.12	0.23<br>+/- 0.027, C	4.9 E-03<br>+/- 6.5 E-03, C		1.7 E-01<br>+/- 0.35 E-01, A
12 hpi	0.93<br>+/- 0.083	0.19<br>+/- 0.026, B/C	3.7 E-02<br>+/- 0.55 E-02, C	1.7 E-03	1.1 E-01<br>+/- 0.097 E-01, A/B
24 hpi	1.18<br>+/- 0.099	0.37<br>+/- 0.051, B/C	7.8 E-02<br>+/- 1.4 E-02, C	5.0 E-04<br>+/- 0.68 E-04, B	9.9 E-02<br>+/- 1.3 E-02, A/B
48 hpi	1.14<br>+/- 0.12	1.15<br>+/- 0.13, A	1.5 E-01<br>+/- 0.13 E-01, A/B	2.9 E-03<br>+/- 0.29 E-03, A	1.6 E-01<br>+/- 0.14 E-01, A

(C)

	WS41	AGO5	AGO8	AGO9	AGO6	AGO7
Mycelium	1.00<br>+/- 0.11	1.3 E-03<br>+/- 0.53 E-03	4.0 E-07<br>+/- 4.5 E-07	3.5 E-04<br>+/- 0.96 E-04, A	1.5 E-05<br>+/- 0.32 E-05	1.8 E-03<br>+/- 0.34 E-03, B
Zoospores	1.36<br>+/- 0.17	1.2 E-04<br>+/- 0.29 E-04	1.1 E-07<br>+/- 0.61 E-07	6.6 E-06<br>+/- 1.5 E-06, B	2.2 E-04<br>+/- 0.39 E-04	1.1 E-01<br>+/- 0.16 E-01, A
Germinated cysts	1.13<br>+/- 0.11	1.0 E-04<br>+/- 0.17 E-04	1.6 E-08<br>+/- 0.94 E-08	1.1 E-05<br>+/- 0.34 E-05, B	1.6 E-05<br>+/- 0.30 E-05	3.5 E-03<br>+/- 0.42 E-03, B
3 hpi	1.19<br>+/- 0.13	2.4 E-03			2.8 E-04	7.6 E-02
6 hpi	1.22<br>+/- 0.12	3.5 E-04		3.9 E-03		6.7 E-02
12 hpi	0.93<br>+/- 0.083			5.3 E-05<br>+/- 4.0 E-05		1.4 E-02
24 hpi	1.18<br>+/- 0.099	5.2 E-05<br>+/- 2.8 E-05		1.0 E-04<br>+/- 0.33 E-04, A/B	2.7 E-06	1.5 E-03<br>+/- 0.31 E-03, B
48 hpi	1.14<br>+/- 0.12	1.7 E-03<br>+/- 0.21 E-03	4.8 E-07	4.7 E-04<br>+/- 1.0 E-04, A	2.1 E-04<br>+/- 0.75 E-04	3.4 E-03<br>+/- 0.55 E-03, B

Supplementary Figure 5. RT-qPCR analysis of AGO and other small RNA biogenesis proteins. Three different life stages and an infection time series (hpi: hours post infection) were assayed in replicate to determine expression levels of (A) DCL1, DCL2, RDR, and (B,C) the AGO homologs in *P. sojae* as compared to housekeeping genes β-tubulin and WS41. The average 2^{-ddCT} +/- SE for each life stage or time series point (versus mycelium) is normalized to the housekeeping gene(s). Tukey groups, when present, are reported. Some early time points did not have detectable transcript levels (empty cells), while others only had one detectable replicate (cells without standard error).

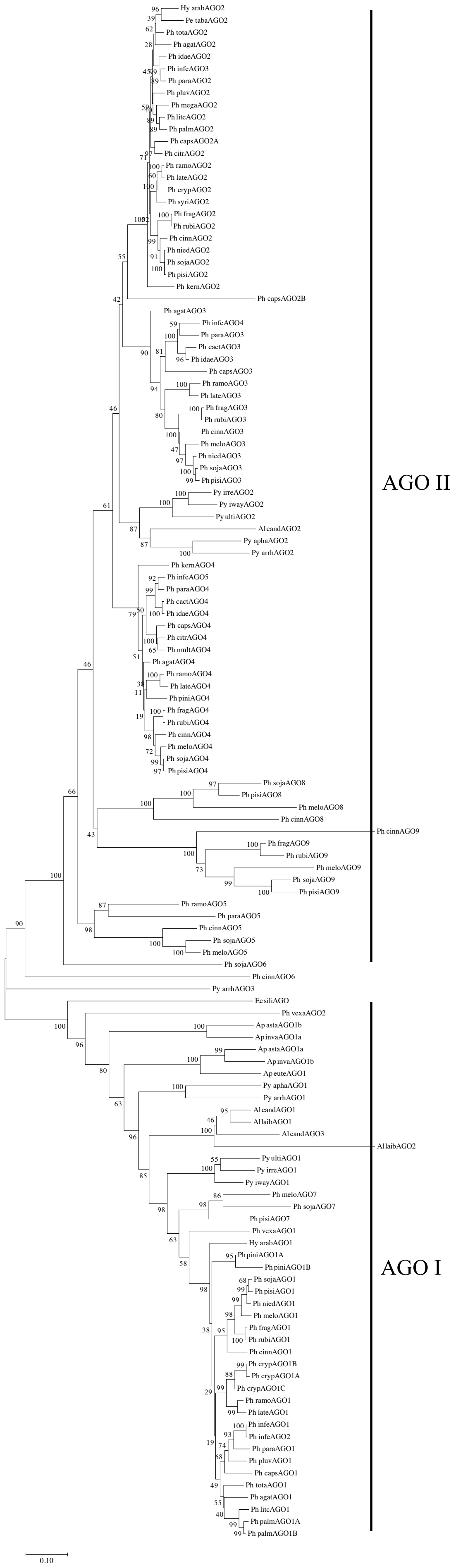

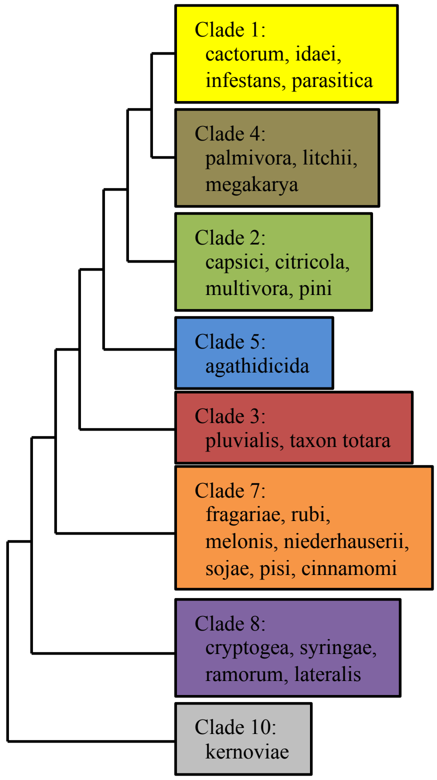

Supplementary figure 6. Phylogram of AGO homologs based on conserved domains. The neighbor-joining tree is based on evolutionary distances computed with the Poisson correction method using pairwise deletion of ambiguous sites. Bootstrap values (10,000 replicates) are indicated, as well as oomycete AGO I and AGO II clades. Species are abbreviated as: Ge spec (Supplementary table 2). Insert is topology of Phytophthora species used in this analysis and clades according to Blair et. al. (2008).

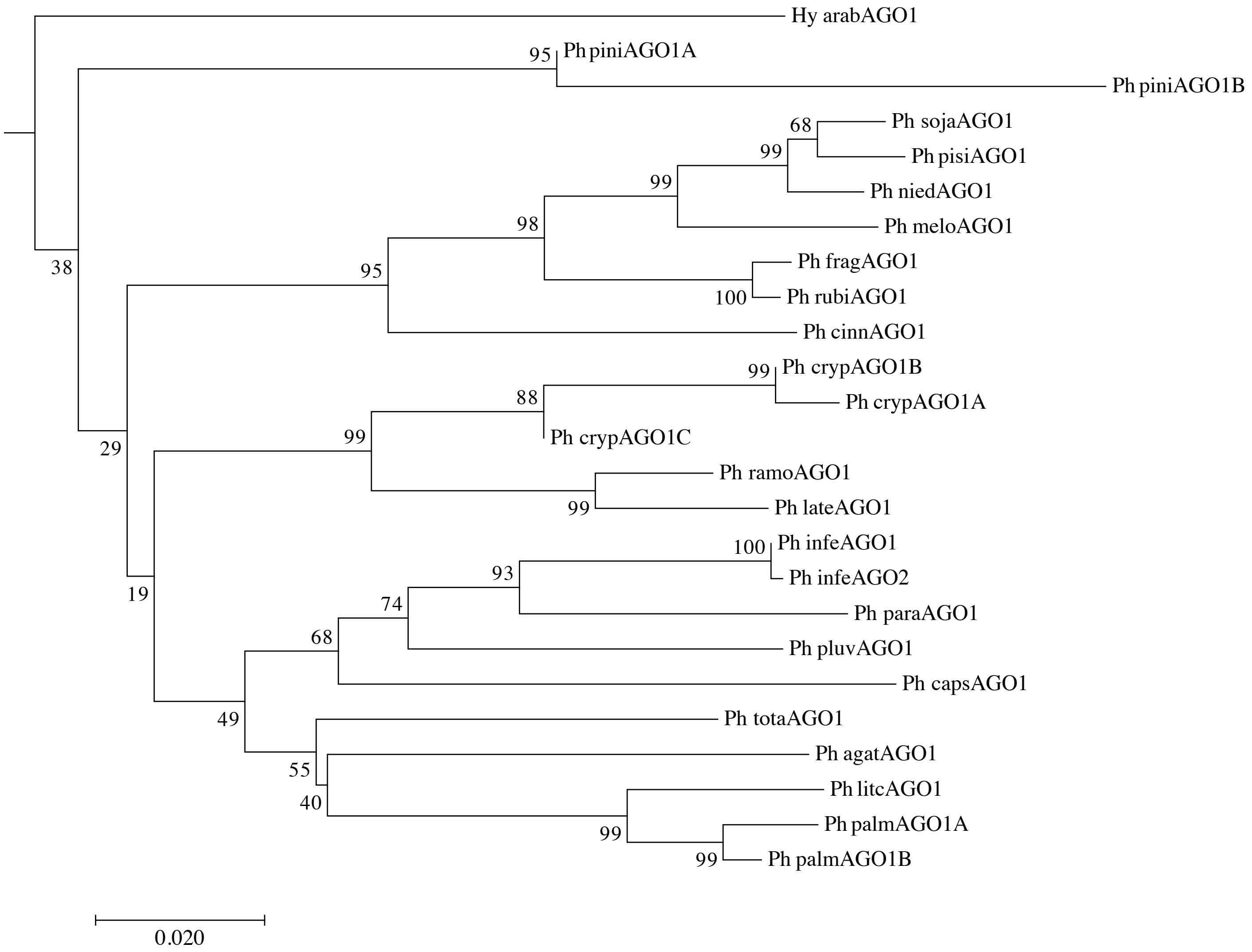

Supplementary figure 7. Phylogram subtree of AGO1 group orthologs based on conserved domains. Bootstrap values are indicated. Species names as in Figure 4.

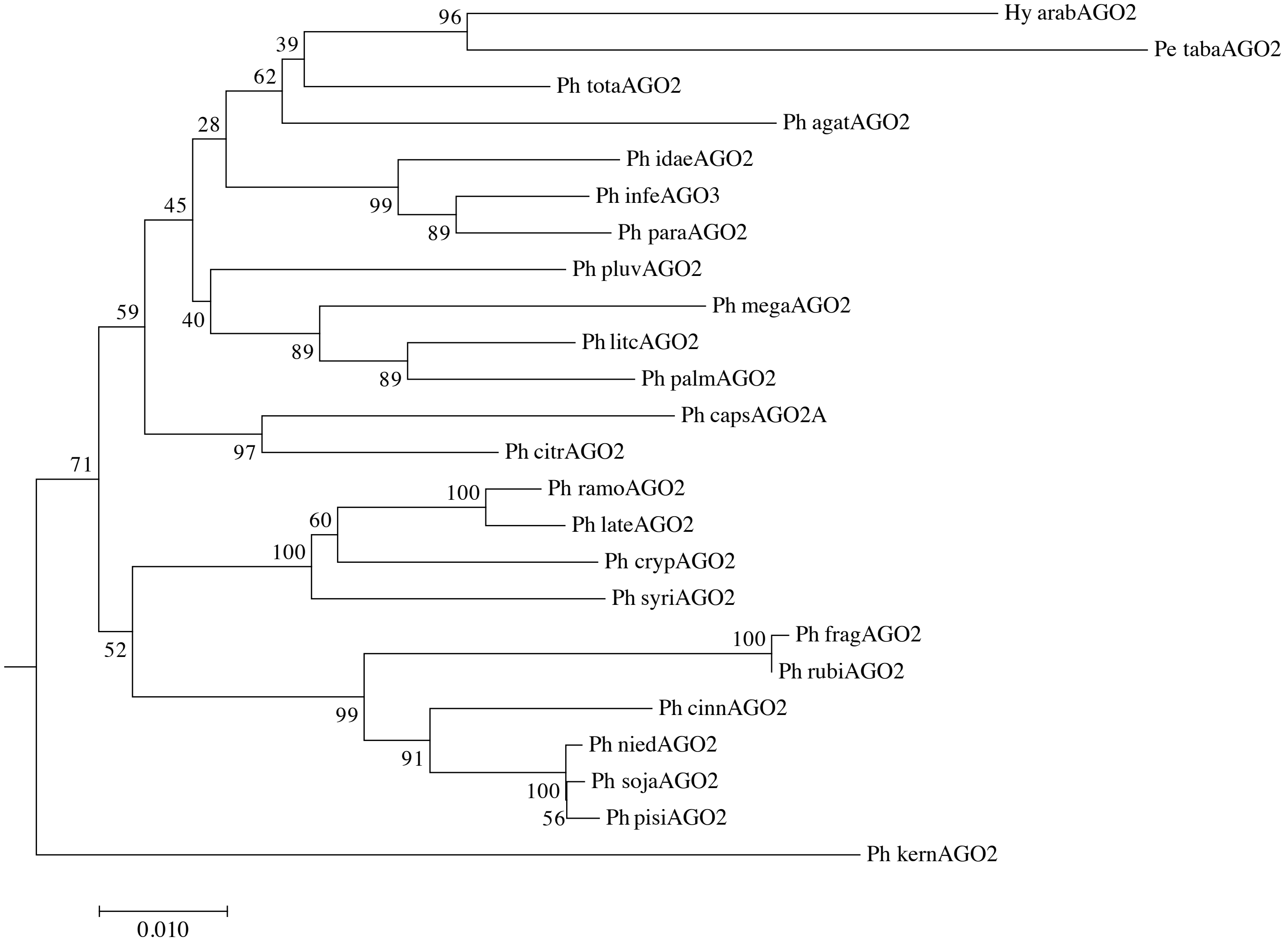

Supplementary figure 8. Phylogram subtree of AGO2 group orthologs based on conserved domains. Bootstrap values are indicated. Species names as in Figure 4.

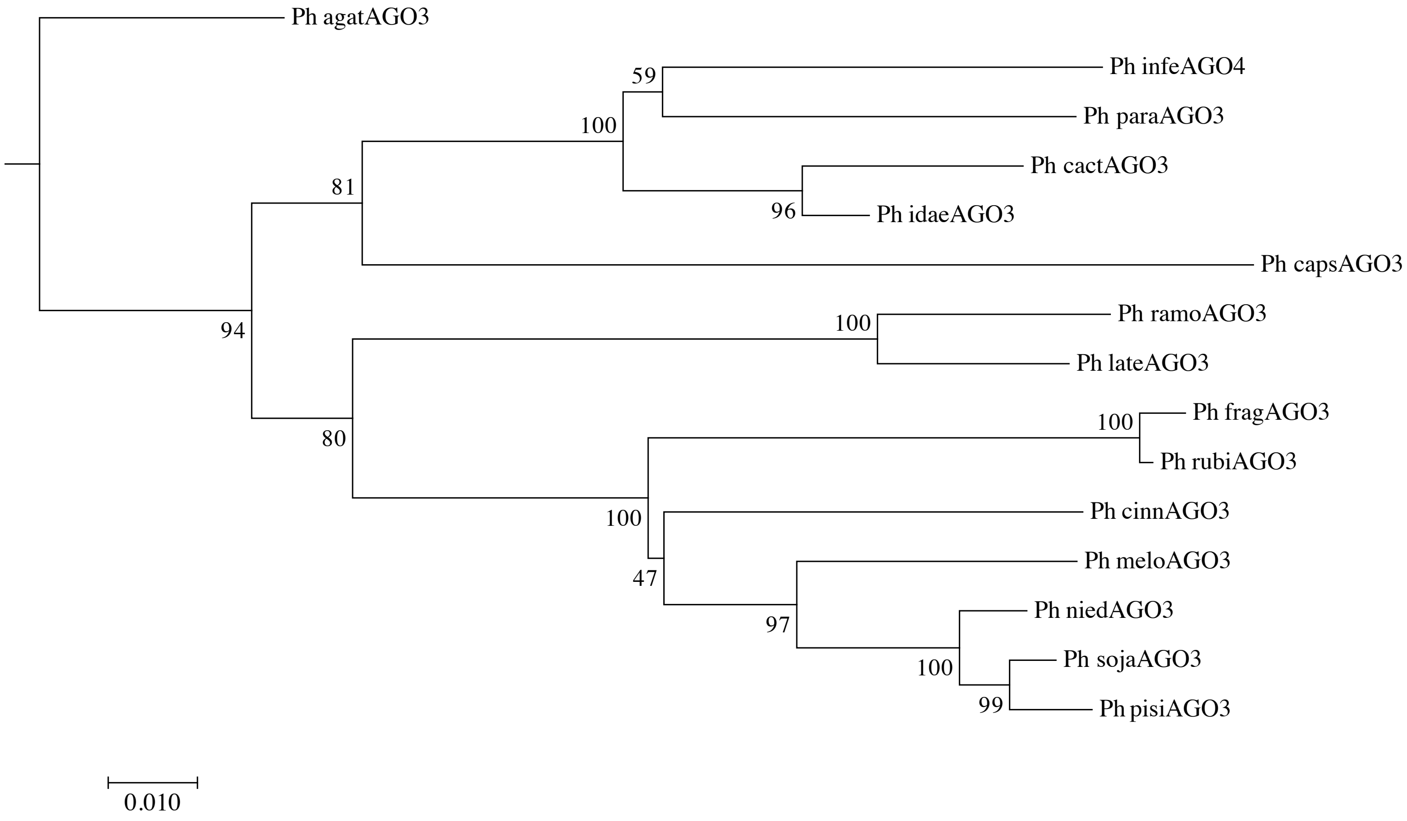

Supplementary figure 9. Phylogram subtree of AGO3 group orthologs based on conserved domains. Bootstrap values are indicated. Species names as in Figure 4.

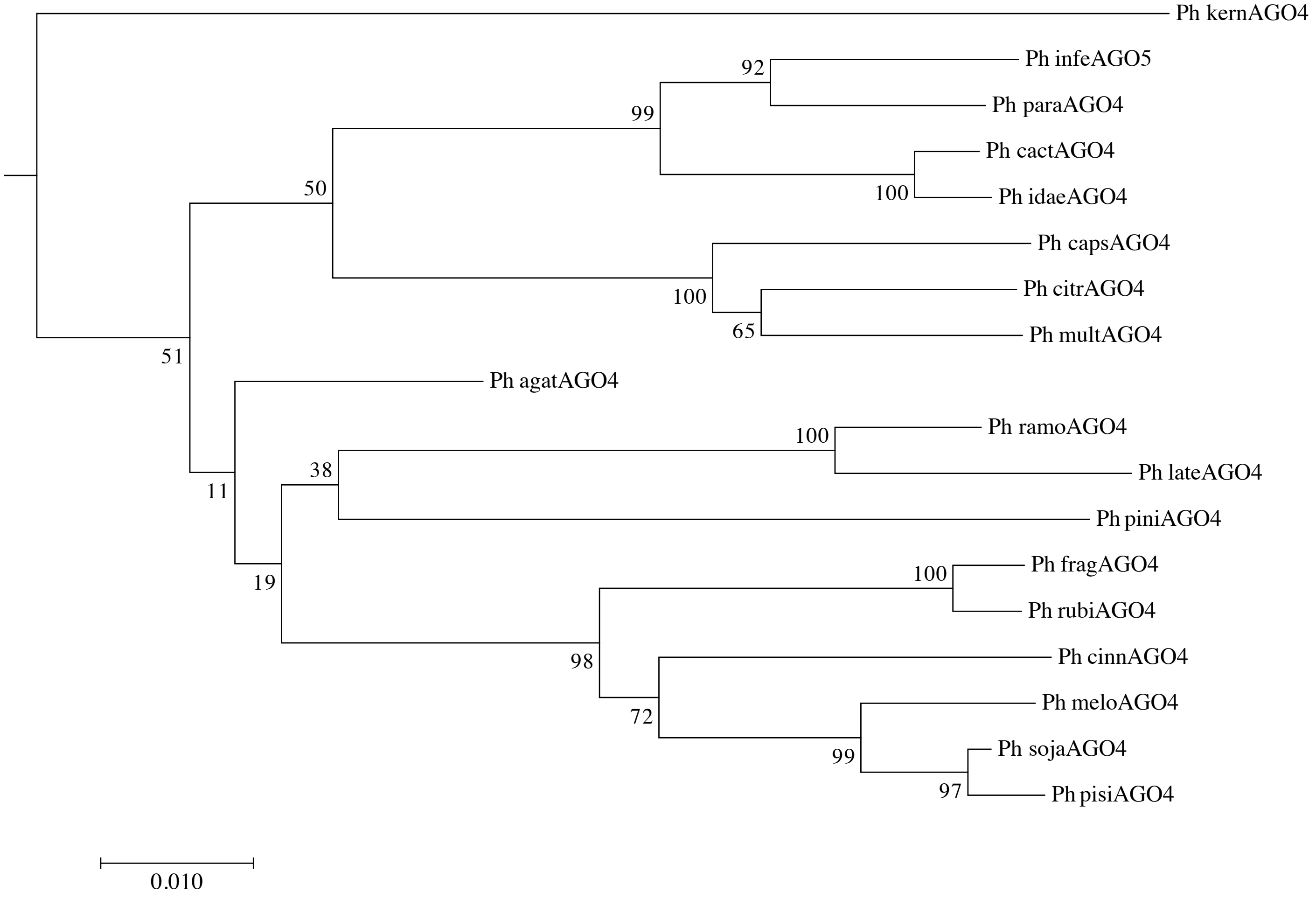

Supplementary figure 10. Phylogram subtree of AGO4 group orthologs based on conserved domains. Bootstrap values are indicated. Species names as in Figure 4.

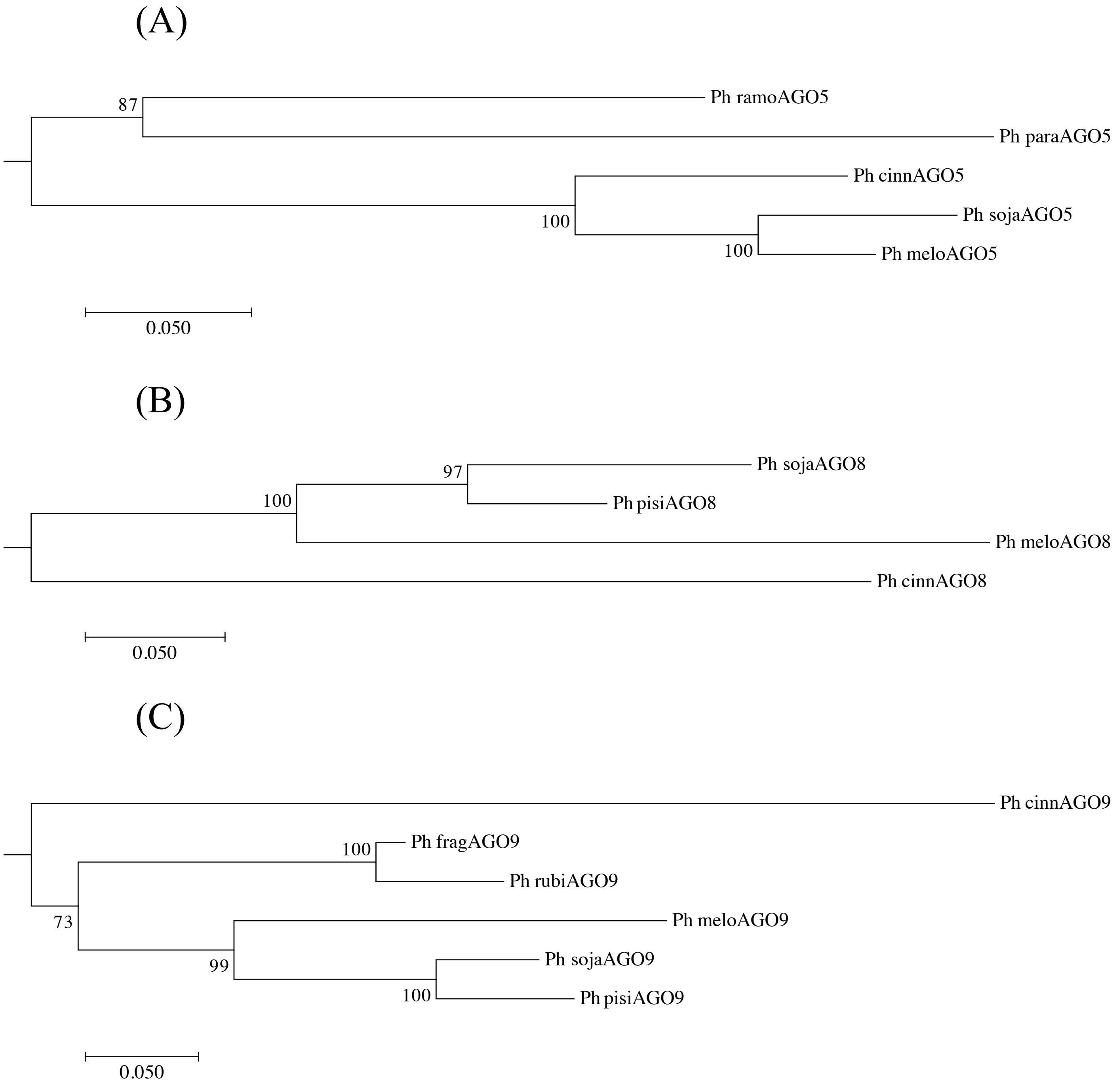

Supplementary figure 11. Phylogram subtrees of additional ortholog groups based on conserved domains. (A) AGO5 group; (B) AGO8 and AGO9 groups. Bootstrap values are indicated. Species names as in Figure 4.
